# Nature vs. nurture: parental care cushions agricultural drought impacts on child health in South Africa

**DOI:** 10.1038/s41598-025-34109-w

**Published:** 2026-01-22

**Authors:** Bopaki Phogole, Dikobe Molepo, Mamadi Theresa Sethusa, Kowiyou Yessoufou

**Affiliations:** 1 Department of Geography, Environmental Management and Energy Studies, University of Johannesburg, Kingsway Avenue, Auckland Park, Johannesburg, South Africa; 2 South African National Biodiversity Institute, Pretoria, South Africa; 3 African Centre for DNA Barcoding, Department of Botany and Plant Biotechnology, University of Johannesburg, Kingsway Avenue, Auckland Park, Johannesburg, South Africa

**Keywords:** Drought, Child health, Low birth weight, Malnutrition, NDVI, SPEI, Environmental sciences, Health care

## Abstract

This study investigates the spatiotemporal trends of drought and its impact on child health in South Africa, focusing on low birth weight (LBW) and severe acute malnutrition in children under five. We collected data on child health indicators (LBW and malnutrition) and social determinants, including orphan status, child food poverty, proximity to clinics, water access, and sanitation access, from the Children’s Institute at the University of Cape Town. Environmental data, comprising the Normalised Difference Vegetation Index (NDVI), Standardised Precipitation Evapotranspiration Index (SPEI), and maximum temperatures, were retrieved from MODIS, Global SPEI, and TerraClimate datasets, covering 2002 to 2022. We then fitted a series of linear regressions and combined them into a structural equation model to explore relationships between socio-environmental factors and child health outcomes. Results indicate that the Northern Cape, Western Cape, and Free State are highly vulnerable to agricultural drought, with NDVI showing a strong negative association with LBW and malnutrition. Orphan status emerged as a stronger predictor of malnutrition than drought. The impact of orphan status on malnutrition level is mediated by limited access to basic services such as water and sanitation. Proximity to clinics significantly influenced access to basic services, highlighting a double burden of healthcare and environmental deprivations. These findings expose the need for targeted interventions to enhance food security, water, and sanitation access, particularly for orphaned children, and to integrate drought mitigation into child health policies in South Africa.

## Introduction

Africa is home to the fastest-growing population in the world, with sub-Saharan Africa (SSA) having the highest population growth rate of 2.5% per year^1^. As a result, SSA is expected to play a major role in global population dynamics. However, the region is threatened by a plethora of socio-environmental challenges that threaten child health and wellness, and thus its future sustainability. SSA has the highest rates of under-5 mortality and child malnutrition in the world^2^. An estimated 1 in 15 children born in SSA die before their fifth birthday, which is 20% greater than the global average^3^. Furthermore, SSA has a 24% prevalence of low birth weight, which is second only to southern Asia^4^. In addition, the prevalence of child malnutrition remains high, primarily driven by high food insecurity and inadequate access to water, sanitation, and healthcare. Around 33.2% of children in SSA are suffering from malnutrition with varying levels of stunting, wasting, and underweight between the different countries in the region^5^. The persistence of the high disease burden in the region is linked to poor access to healthcare services, among others. It is estimated that only 42.6% of reproductive-age women have access to health care services^6^, with variations depending on the degree of urbanisation^7^.

To compound the challenges related to the disease burden, SSA is vulnerable to the most severe impacts of environmental disasters such as drought^8^. Although slow to evolve and persistent, drought is considered the most far-reaching natural disaster^9^ and a major contributor to climate-related health effects^10^. Recent projections estimate that 5% − 8% of the SSA region will experience increasing drought intensity, resulting in the aridification of over 43% of agricultural land in the next decade^11^. Moreover, climate change is expected to further accelerate the drought conditions and their impacts on food security, water availability and economic impacts^12^. These could, in turn, increase the prevalence of environment-linked diseases^13^, thus compounding the pressure on the already strained and limited health system in the region.

Existing studies show that exposure to drought is linked to a reduction in birth weight^14–16^. In Nepal, exposure to drought in the first trimester is linked to a reduction of 82.9 g in birth weight^16^. Similarly, drought exposure in the second trimester is associated with a 87-gram reduction in birth weight in Vietnam^14^. In the context of Africa, a similar study in Sierra Leone reports a 7.9% increase in the proportion of low birth weight in response to drought exposure^15^. Drought exposure is also linked to the incidence of child malnutrition in Kenya^17^, Ethiopia^18^, and other low and middle-income countries^19^. Moreover, prolonged and severe drought conditions have been linked to increased infant mortality risks across Africa^20^. In particular, existing evidence indicates that child mortality rises as drought conditions worsen in Southern Africa, highlighting the vulnerability of children to climate-related shocks^21^.

Pre-natal exposure to drought has been found to significantly increase the risk of under-five child stunting, with the most pronounced effects observed during the second and third trimesters in 32 low- and middle-income countries^22^. Drought conditions are also associated with higher infant mortality, lower birth weight, and long-term socioeconomic disadvantages such as reduced income and poor housing conditions^23^. In addition, drought diminishes the availability of essential nutrients such as calories, protein, and zinc and reduces overall diet diversity, thereby negatively impacting child health and increasing the prevalence of stunting among rural children in countries such as Uganda^24^. Evidence further suggests that agricultural drought increases the risk of child undernutrition, particularly in communities where crop farming predominates across sub-Saharan Africa^18^. Undernutrition itself has been identified as a major risk factor for infectious and respiratory diseases, which continue to be the leading causes of child mortality^24,25^. In addition, evidence indicates that children are particularly susceptible to the combined nutritional and infectious disease burdens exacerbated by drought, due to their high dependency on adequate food intake for proper growth and development^26^.

The drought-health nexus is driven by, *inter alia*, hydrological shocks that significantly reduce agricultural productivity and food security, water-related illnesses such as cholera and amoebiasis^13^, and heat stress^27^. A recent large-scale study reported a strong and consistent link between prolonged droughts and an increased risk of diarrhoea among children under five across 51 low- and middle-income countries, with greater effects observed in households with limited access to water, soap for handwashing, or where water collection required more time^28^. Similarly, evidence from South Africa indicates that during the most extended drought periods, children exhibit greater vulnerability, with significantly high mortality risks across multiple specific causes of death^29^.

The current study contributes to this knowledge by exploring the association between drought and child health using South Africa as a case study. South Africa is classified as a semi-arid country, but with some regions categorised as arid^30^. Furthermore, despite its economic status as an upper-middle-income country in SSA, South Africa still has substantial incidences of low birth weight and child malnutrition^31^. Further, deprivations in access to water, sanitation, and health care, all of which have been linked to poor child health, are still prevalent in the country^32^. This makes South Africa an ideal case study for investigating the impact of drought on the prevalence of low birth weight and malnutrition in the context of developing regions.

## Results

### The Spatiotemporal dynamics of child wellness and drought indices

**Fig. 1:**
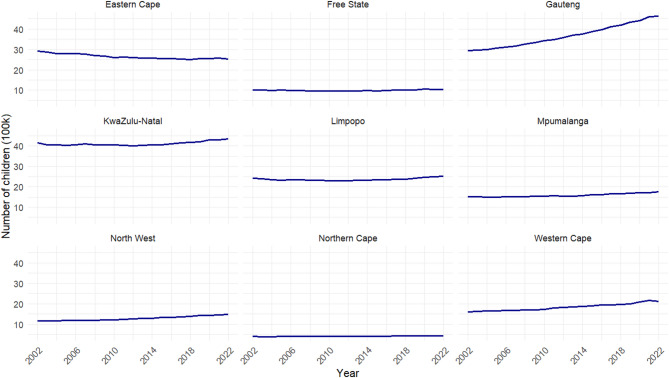
The number of children in each province between 2002 and 2022.

There is a discernible variation in the population of children across the nine provinces, with the highest population in Gauteng and the lowest in the Northern Cape (Fig. 1). Similarly, there is a strong variation in the population trends between 2002 and 2022. In some provinces, e.g., Free State, Limpopo, and Northern Cape, the total number of children is generally consistent during the study period. Contrastingly, some provinces witness an increasing trend, e.g., Gauteng, KwaZulu-Natal, Mpumalanga and Western Cape, whereas the Eastern Cape is the only province witnessing an overall declining population of children.

**Fig. 2:**
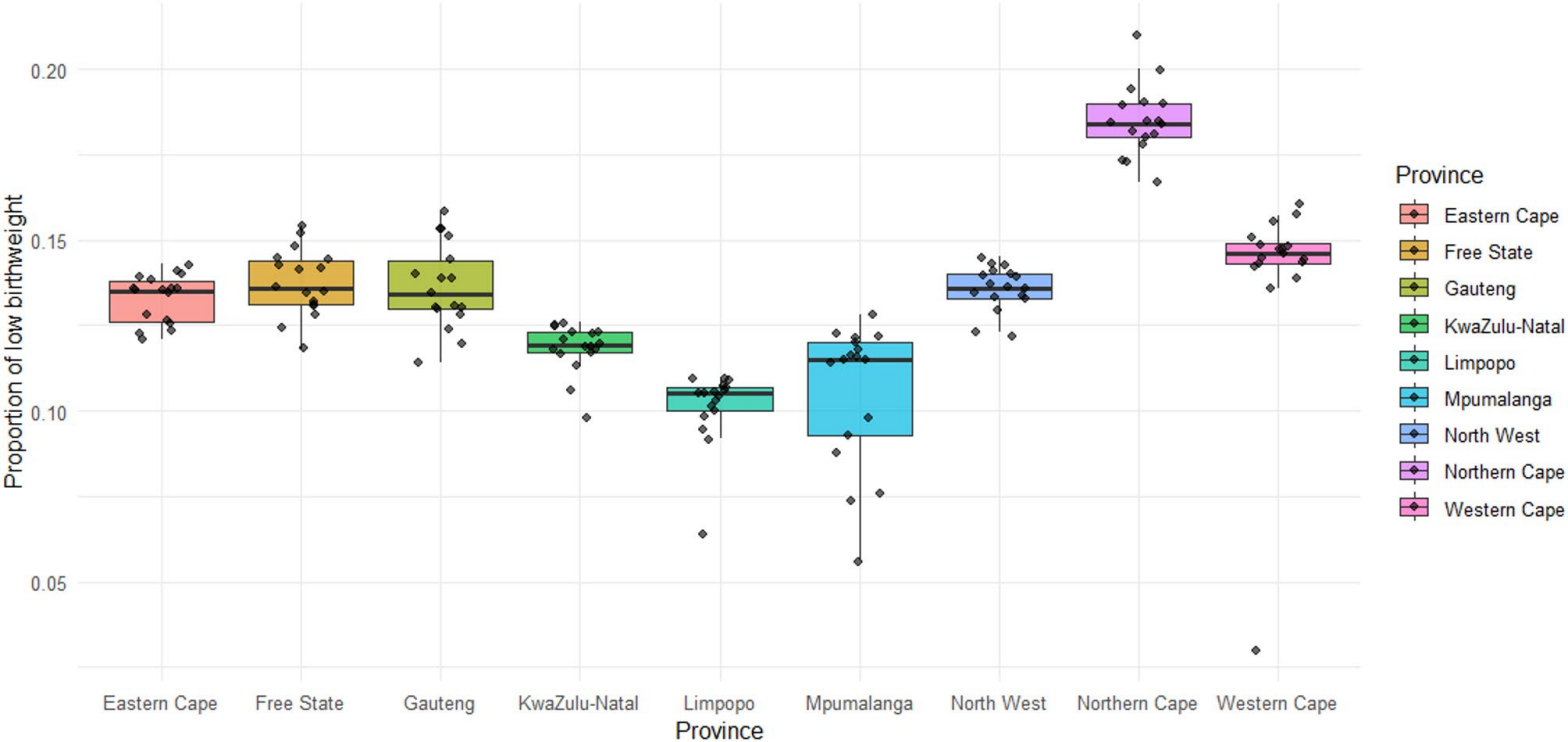
A comparison of the overall prevalence of LBW between the provinces.

**Fig. 3:**
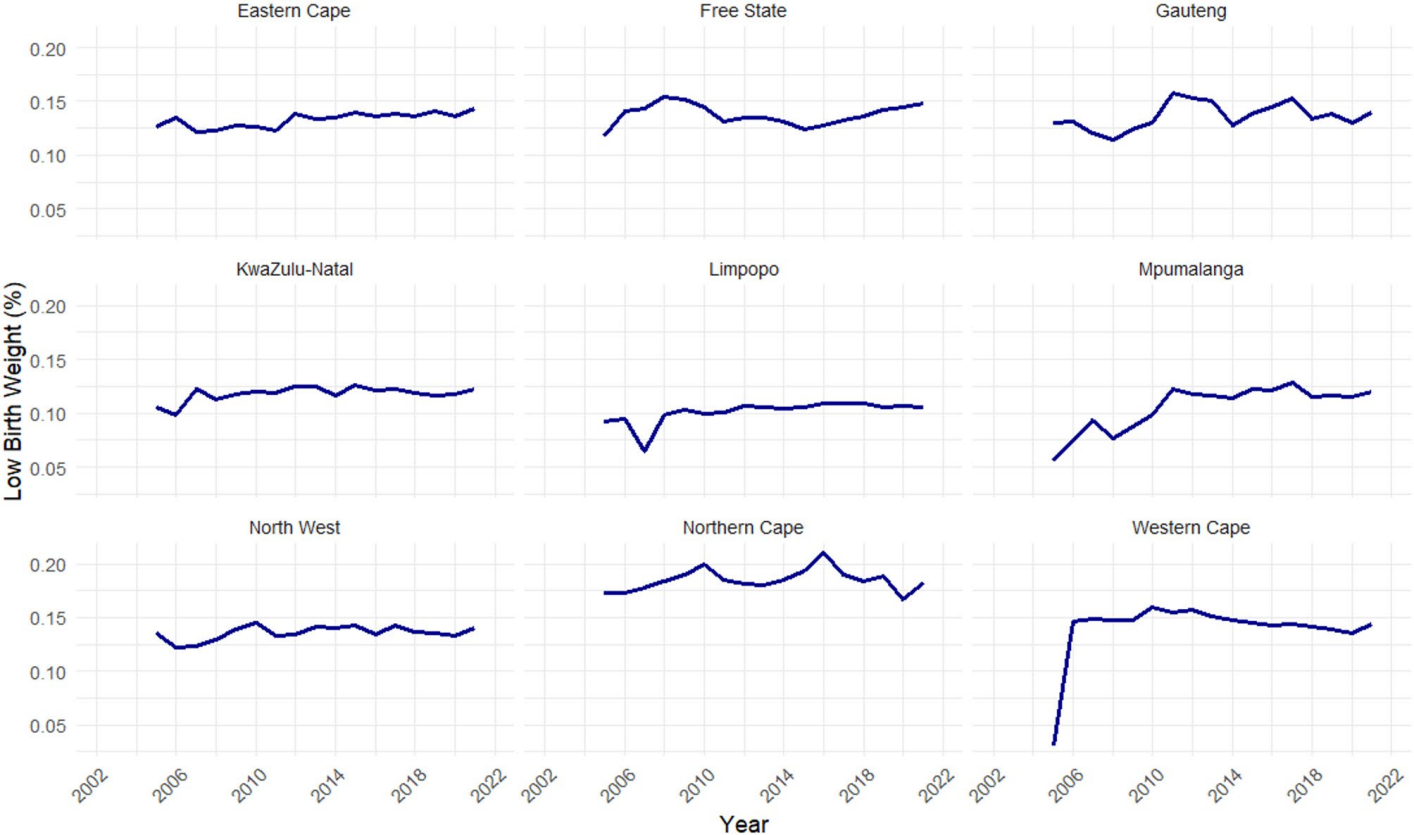
The prevalence of low birth weight (LBW) from 2002 to 2022, stratified by province.

All the provinces have a moderately low prevalence of LBW, except the Northern Cape (Fig. 2). Furthermore, most provinces, i.e., Eastern Cape, Limpopo, North West, Gauteng, and KwaZulu-Natal, experienced minor fluctuations in the proportion of LBW throughout the study period (i.e., 2002 to 2022) (Fig. 3). Only Mpumalanga and, to a larger extent, Western Cape experienced a sharp increase in the prevalence of LBW from 5% to 3% in 2005 to 12% and 14% in 2021, respectively.

**Fig. 4:**
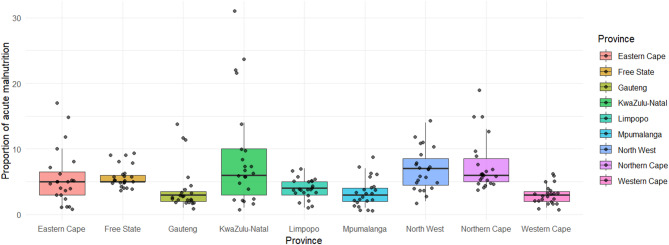
The prevalence of severe acute malnutrition per province in South Africa.

**Fig. 5:**
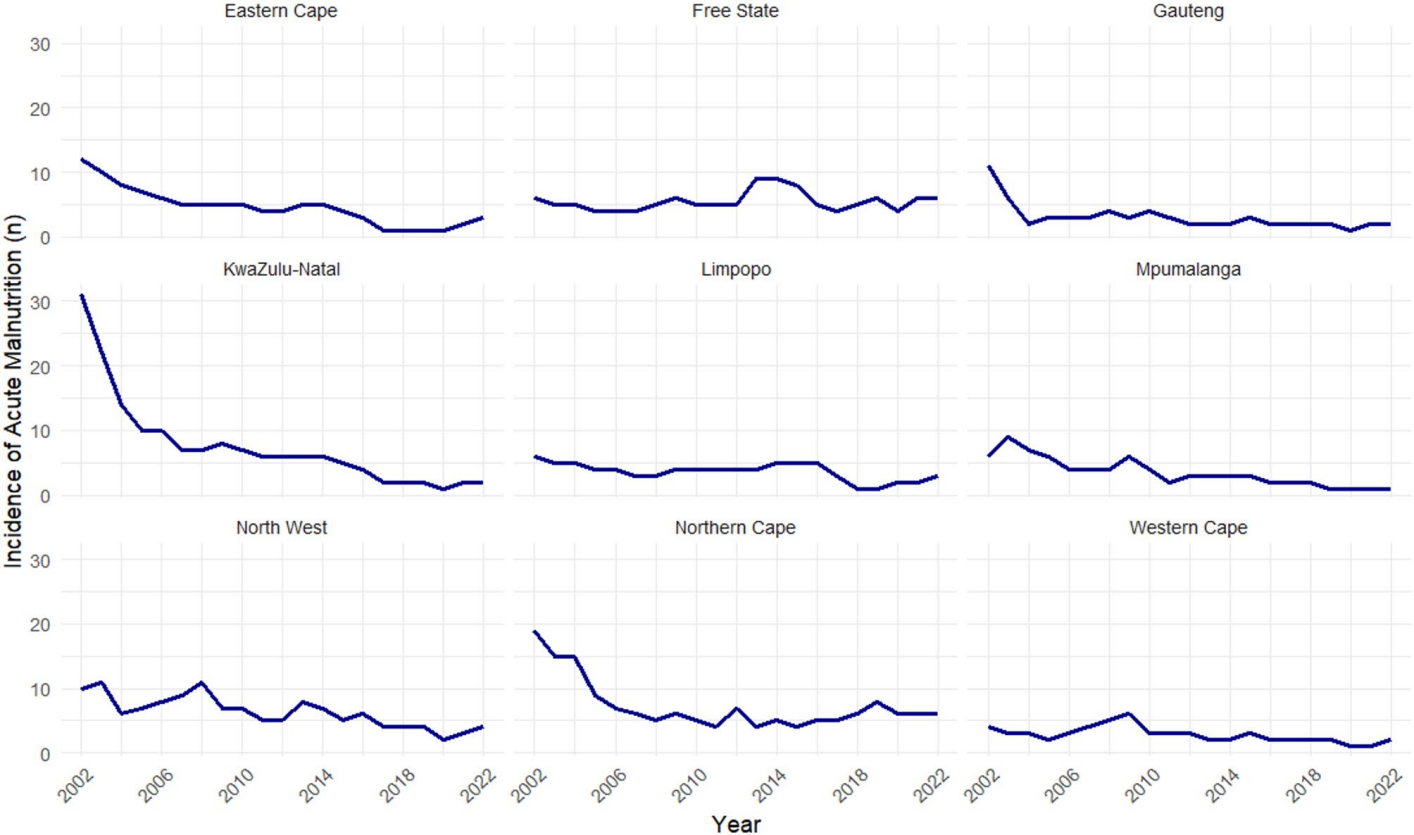
The prevalence of severe acute malnutrition from 2002 to 2022, stratified by province.

The national trends of the prevalence of acute malnutrition are showing a low and steady trend in most of the provinces (Fig. 4). Historically, KwaZulu-Natal (*n* = 31), Northern Cape (*n* = 19), and Eastern Cape (*n* = 12) provinces had the highest prevalence of malnutrition from a population of 4.15 million, 0.39 million, and 2.92 million children, respectively (Fig. 5). All three provinces showed sharp declines around the year 2004. In contrast, Limpopo, North West, and Western Cape consistently experienced low prevalence of acute malnutrition throughout the study period, with an overall declining trend. The incidences of acute malnutrition in the Free State remained stable through the study period, with no obvious increasing or decreasing trend.

### Structural equation modelling of the socio-environmental predictors of low birth weight and malnutrition

**Fig. 6:**
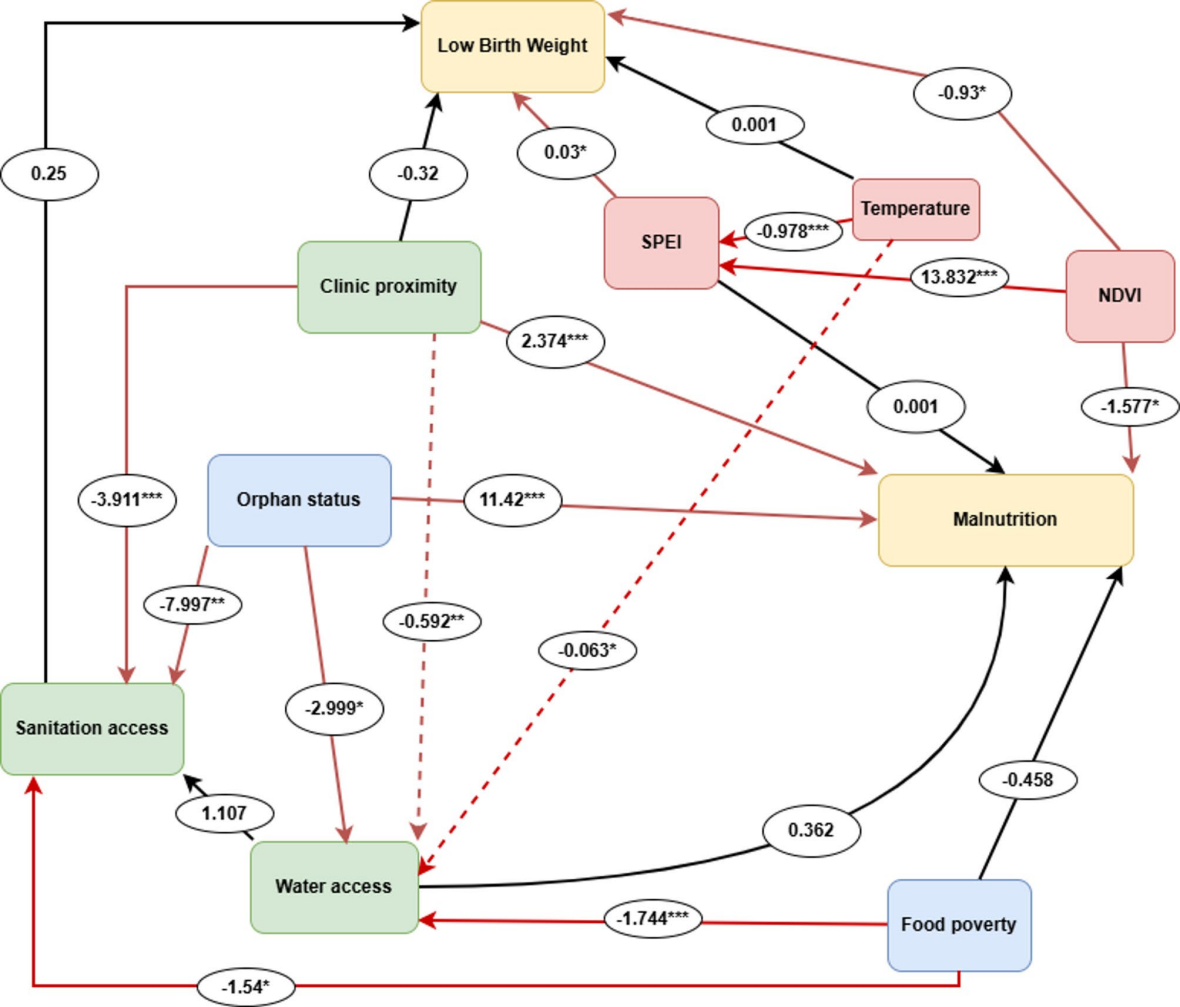
A structural equation model of the socio-environmental predictors of low birth weight and acute malnutrition in South Africa. Red lines indicate statistically significant paths (*p* < 0.05), and broken lines are used for visual enhancement. * *P* < 0.05; ** *p* < 0.01; *** *p* < 0.001.

The model showed a good fit for our data (Fisher’s C = 31.709; *p* = 0.134). Low birth weight is strongly associated with NDVI and SPEI (Table 1; Fig. 6). NDVI, which denotes agricultural drought, has a negative association with LBW (β=−0.931 ± 0.38, *p* = 0.014), whereas hydrological drought, indicated by SPEI, has a positive association with LBW (β = 0.032 ± 0.01, *p* = 0.012). NDVI is the only measure of drought with a strong link to the prevalence of acute malnutrition (β=−1.577 ± 0.61, *p* = 0.01). Furthermore, acute malnutrition is strongly predicted by social factors such as clinic proximity (β = 2.341 ± 0.65, *p* < 0.001) and orphan status (β = 11.42 ± 3.55, *p* = 0.001). Sanitation access is negatively associated with clinic proximity (β=−3.911 ± 0.40, *p* < 0.001), food poverty (β=−1.54 ± 0.76, *p* = 0.044), and orphan status (β=−7.997 ± 2.54, *p* = 0.002). Water access is moderately associated with clinic proximity (β=−0.592 ± 0.2, *p* = 0.003), orphan status (β=−2.999 ± 1.44, *p* = 0.037), and temperature (β=−0.063 ± 0.03, *p* = 0.046), and strongly associated with food poverty (β=−1.744 ± 0.38, *p* < 0.001).

**Fig. 7:**
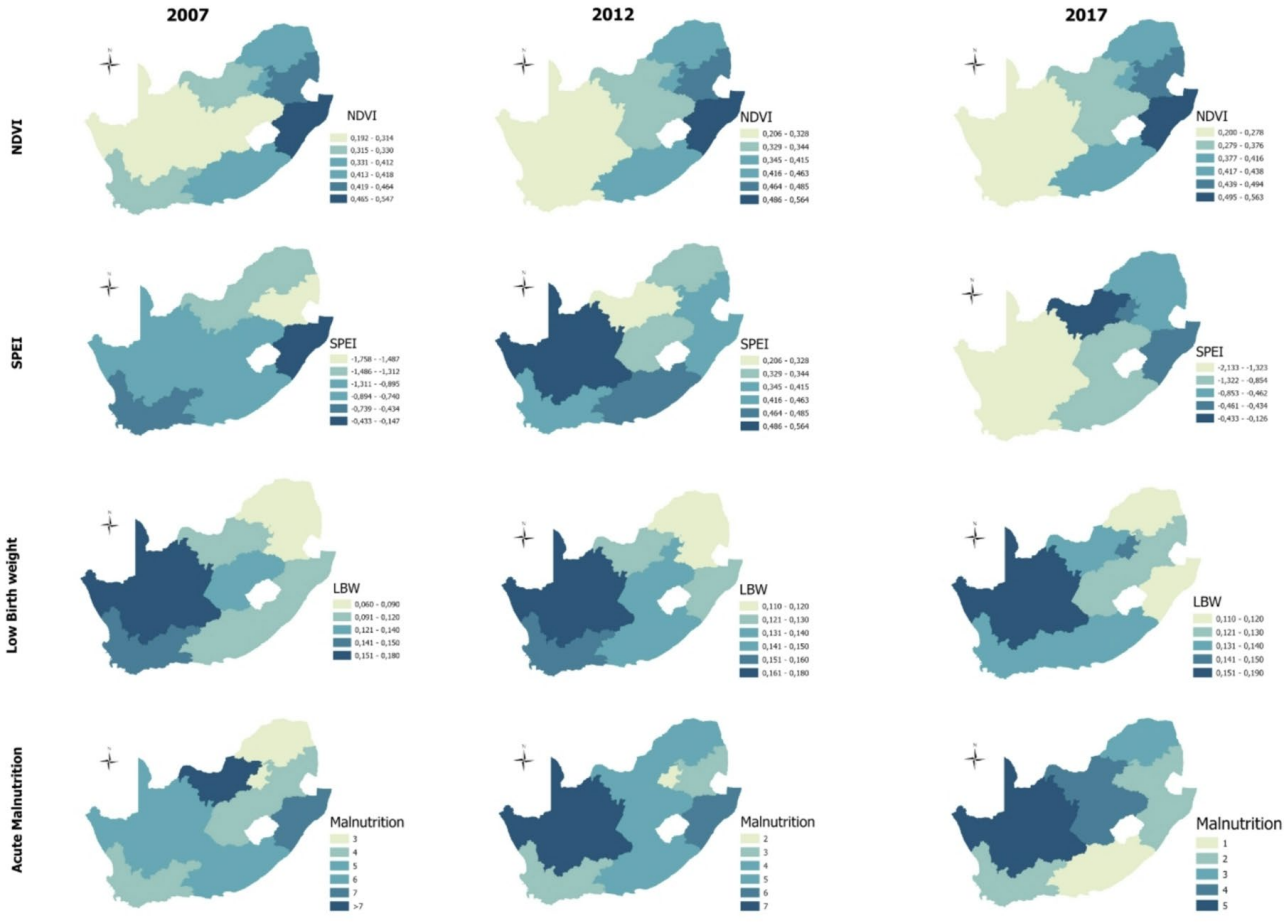
Geospatial illustration of normalised vegetation index (NDVI), standardised precipitation evapotranspiration index (SPEI), low birth weight (LBW), and acute malnutrition for the periods 2007, 2012, and 2017.

Figure 7 depicts the spatial distribution of the strongly correlated drought and socio-environmental indicators. Throughout the study period, NDVI is greater in the eastern parts of the country, and it is inversely matched by LBW and malnutrition trends, which are consistently greater in the western parts of the country. In contrast, the spatial distribution of SPEI varies throughout the study period. In 2007 and 2012, the highest SPEI values were concentrated in the south-western parts of the country, which shifted to the north-eastern parts in 2017.

**Table 1:** A summary of the structural equation model of the socio-environmental predictors of low birth weight and acute malnutrition.

Response	Predictor	Estimate (β)	SE (±)	DF	Critical value	*P*-value
Low birth weight (*n* = 153)	SPEI (*n* = 189)	0.032	0.01	135	2.5	0.012*
Low birth weight (*n* = 153)	NDVI (*n* = 189)	−0.931	0.38	135	−2.845	0.014*
Low birth weight (*n* = 153)	Clinic proximity (*n* = 171)	−0.32	0.18	135	−1.80	0.07
Low birth weight (*n* = 153)	Sanitation access (*n* = 171)	0.25	0.14	135	1.72	0.085
Low birth weight (*n* = 153)	Temperature (*n* = 189)	0.001	0.02	135	0.0318	0.975
Malnutrition (*n* = 189)	SPEI (*n* = 189)	0.001	0.05	135	0.02	0.983
Malnutrition (*n* = 189)	NDVI (*n* = 189)	−1.577	0.61	135	−2.58	0.01**
Malnutrition (*n* = 189)	Water access (*n* = 189)	0.362	0.52	135	0.69	0.49
Malnutrition (*n* = 189)	Food poverty (*n* = 180)	−0.458	0.94	135	−0.49	0.624
Malnutrition (*n* = 189)	Orphan status (*n* = 189)	11.42	3.55	135	3.21	0.001***
Malnutrition (*n* = 189)	Clinic proximity (*n* = 171)	2.374	0.65	135	3.65	0.000***
Water access (*n* = 189)	Food poverty (*n* = 180)	−1.744	0.38	135	−4.54	0.000***
Water access (*n* = 189)	Clinic proximity (*n* = 171)	−0.592	0.20	135	−2.93	0.003**
Water access (*n* = 189)	Temperature (*n* = 189)	−0.063	0.032	135	−1.99	0.046*
Water access (*n* = 189)	Orphan status (*n* = 189)	−2.999	1.44	135	−2.08	0.037*
Sanitation access (*n* = 171)	Water access (*n* = 189)	1.107	0.72	135	1.54	0.123
Sanitation access (*n* = 171)	Clinic proximity (*n* = 171)	−3.911	0.4	135	−9.68	0.000***
Sanitation access (*n* = 171)	Orphan status (*n* = 189)	−7.997	2.54	135	−3.14	0.002**
Sanitation access (*n* = 171)	Food poverty (*n* = 180)	−1.54	0.76	135	−2.02	0.044*
SPEI (*n* = 189)	Temperature (*n* = 189)	−0.978	0.11	135	−8.62	0.000***
SPEI (*n* = 189)	NDVI (*n* = 189)	13.832	2.53	135	5.47	0.000***

† n = number of observations; DF = degree of freedom; SE = Standard error; * *P* < 0.05; ** *p* < 0.01; *** *p* < 0.001

## Discussion

### Spatiotemporal trends of drought and child health in South Africa

The spatiotemporal illustration of drought distribution in South Africa reveals that the Western Cape, Free State, and, to a greater extent, the Northern Cape are the most vulnerable regions to agricultural drought. In particular, the Northern Cape has the lowest NDVI values for 2007, 2012, and 2017, when compared to the other eight provinces. The vulnerability of the Northern Cape to drought has been extensively documented in the literature^33,34^. Although South Africa is largely classified as a semi-arid country, some regions are considered arid, most of which are contained in the Northern Cape^35^. Studies show that the Northern Cape is most vulnerable to a variety of droughts, including hydrological and agricultural drought^34^. The persistent drought conditions in the province are linked to myriad socioeconomic and environmental consequences, including economic losses, reduced agricultural productivity, and water shortages. Ultimately, these challenges lead to reduced food security in the province. The impacts of drought, however, are not limited to the Northern Cape province; they are distributed across other provinces throughout the country, albeit with varying degrees of occurrence, severity, and impacts^36^.

With regard to the distribution of LBW and malnutrition, there are discernible differences in trends between the provinces over time. LBW consistently features more in the western provinces, such as the Northern Cape and Western Cape, for 2007, 2012, and 2017. The distribution of malnutrition is less consistent, with high occurrence in North West and Mpumalanga in 2007, then shifting to the Northern Cape in 2012, and a cluster of Northern Cape, North West, and Free State appearing in 2017. Although there are no longitudinal studies on the nationwide geographical distribution of these health indicators, cross-sectional regional studies are reporting substantial occurrences of LBW or child malnutrition in some parts of Gauteng^37^ and KwaZulu-Natal^38,39^, and Limpopo^40^. The dynamism of the prevalence of LBW and malnutrition can be attributed to the myriad socio-environmental driving factors, which may vary between provinces over the years. Importantly, the current study aimed to investigate the impact of drought as one of the temporally and spatially variable determinants of LBW and malnutrition in South Africa.

### Socio-environmental predictors of child health

NDVI and SPEI are the only measures of drought that are strongly correlated with indicators of child health. NDVI, a measure of agricultural drought, is strongly and negatively associated with LBW and malnutrition, whereas SPEI is strongly and positively correlated with low birth weight. This implies that increased agricultural drought, denoted by low NDVI values, is linked to increased occurrence of low birth weight and severe acute malnutrition. The influence of agricultural drought on child health is not unique to our study. For example, existing study reports an increase of 7.9% in the proportion of LBW during agricultural harvest drought^15^. Furthermore, a systematic review shows that increased drought exposure is positively linked to undernutrition^19^. The strong link between agricultural drought and child health can be explained by the decline in agricultural crop yield, economic hardships, and maternal malnutrition, which are strongly linked to drought incidence^16,41,42^. However, our findings on the association between SPEI and LBW do not conform to existing evidence. In our study, we found a positive association between SPEI and LBW, implying an increased proportion of LBW during wet conditions. This exposes the intricate dimensions of the different forms of drought and how they have varying effects on child health and wellness. Furthermore, this may suggest that other socio-environmental factors may be key to defining children’s vulnerabilities to the impacts of hydrological droughts.

Indeed, our investigation of the social determinants of child health shows that orphan status, an indicator of inadequate parental care^43^, is linked to a greater incidence of malnutrition. These results outline the importance of parental care in achieving adequate nutrition for children, mirroring other studies^44,45^. A recent study in South Africa found that orphans are more likely to suffer from undernutrition primarily due to limited access to water and sanitation facilities^46^. Orphan status is also strongly associated with sanitation access and water access in our study. This confirms orphanage as a key predictor of water and sanitation deprivation among children in South Africa. The strong association between orphanage and access to basic services underscores the role of parental care in moderating the harsh socio-environmental impacts, including drought, on children. South Africa has made significant strides in supporting new mothers and children in foster care through various grant mechanisms that aim to improve child and maternal health^47^. The proposed Maternal Support Grant Draft Policy aims to support expectant mothers from the first trimester to the first 3 years of a child’s life^48^, and the child support grant that forms part of the government social assistance programme^47^. However, our results suggest that these child support initiatives need to be supplemented by targeted interventions to improve access to adequate food, water, and sanitation services, especially among orphaned children. Furthermore, environmental stressors such as droughts need to be an integral part of policies and programmes to support and protect newborn and orphaned children.

Water access and sanitation access are negatively predicted by proximity to clinics, with access improving for children staying near clinics. This is a reflection of a double burden of limited access to health care, water and sanitation^49,50^. The inadequate access to water and healthcare facilities is a regional issue that extends beyond South Africa. A regional study of 19 sub-Saharan cities found that only 33% of the population lives within 15 min of hospitals, with worse access for populations in informal areas^51^. Similarly, access to water and sanitation in the sub-Saharan region remains low, with devastating impacts in lower-income regions^52^. Therefore, given its rapidly rising population and susceptibility to climate change-induced drought, the sub-Saharan region is likely to see deeper deprivations in water and sanitation services, with severe impacts on child health and wellbeing. Furthermore, the observed relationship between drought, temperature, water access confirms the vulnerability of drought-stricken communities to extreme water deprivation^53,54^. Therefore, there is an urgent need to address deficiencies in water and sanitation infrastructure, and the accessibility of healthcare facilities. Furthermore, orphaned children need to be prioritised as one of the most vulnerable demographics to environmental shocks.

## Conclusions

This study provides insights into the intricate relationship between drought, socio-environmental factors, and child health outcomes in South Africa, with significant implications for policy and intervention strategies. The Northern Cape, Western Cape, and Free State provinces are most vulnerable to severe agricultural drought. These drought conditions are strongly associated with increased incidences of LBW and malnutrition. However, the positive correlation between SPEI and LBW suggests higher LBW during wetter conditions. This observation goes against prevailing evidence in other regions and underscores the complex and varying impacts of different drought types on child health. Therefore, further investigation into mediating socio-environmental factors of hydrological drought on child health is needed, especially in the context of Africa and other developing regions.

Beyond environmental stressors, social determinants such as orphan status play a critical role in child health. In particular, we found a stronger influence of social determinants (i.e., orphan status) on malnutrition than drought. Orphaned children face compounded vulnerabilities, including limited access to water, sanitation, and adequate nutrition, highlighting the pivotal role of parental care in child health and well-being. Proximity to clinics also significantly predicts access to water and sanitation, revealing a double burden of inadequate healthcare and basic services. These challenges are not unique to South Africa but reflect broader regional issues in sub-Saharan Africa where population growth and climate change exacerbate deprivations in water, sanitation, and healthcare access.

To address these challenges, South Africa’s child and maternal support mechanisms, such as the proposed Maternal Support Grant Draft Policy and the Child Support Grant, should be strengthened with targeted interventions. These should prioritise improving access to food, water, and sanitation for vulnerable groups, particularly orphaned children, and integrate environmental stressors like drought into policy frameworks. Furthermore, investments in water and sanitation infrastructure, alongside improved access to healthcare facilities, are critical to mitigating the adverse effects of drought and socio-environmental deprivations on child health. By addressing these interconnected issues, policymakers can enhance the resilience of South Africa’s children to environmental and social challenges and facilitate the improvement of child health outcomes and long-term well-being.

## Methodology

### Data sources

We relied on the Children’s Institute for data on child health and social health predictors, and satellite-derived indices for environmental data. Children’s Institute (http://childrencount.uct.ac.za/index.php) is based at the University of Cape Town, and it consolidates data on child health and wellness from a diversity of national surveys and administrative data. Environmental data, relating to drought, were retrieved from the Moderate Resolution Imaging Spectrometer (MODIS), Global SPEI, and TerraClimate datasets using the Google Earth Engine Platform (https://earth.google.com/web/). A summary of the data collected is presented in Table 2. All the data were extracted at the provincial level to match how the child health data are reported.

**Table 2:** A summary of the source and description of the data used in this study.

Variable	Indicator	Description	Source
Low birth weight	Child health	The proportion of children who weigh less than 2.5 kg at birth	Children’s Institute database
Severe acute malnutrition	Child health	The number of under-5 children newly diagnosed with severe malnutrition per 1000 under-5 children. Severe malnutrition is measured by the child’s weight in relation to their height^55^	Children’s Institute database
Clinic proximity	Health services	The proportion of children living far from health facilities.	Children’s Institute database
Sanitation access	Health services	The proportion of children who reside in a household with access to basic sanitation	Children’s Institute database
Water access	Health services	The proportion of children with access to safe and reliable drinking water at home	Children’s Institute database
Orphan status	Parental care	The proportion of children without both maternal and paternal parents	Children’s Institute database
Food poverty	Economic depravation	The proportion of children who reside in income-poor households	Children’s Institute database
Normalised difference vegetation index (NDVI)	Agricultural drought	A metric (ranging from − 1 to 1), that quantifies vegetation health and density.	MODIS
Standardised precipitation evapotranspiration index (SPEI)	Hydrological drought	A metric of drought conditions measured as deviations of precipitation and evapotranspiration (accumulated over the previous 12 months) from the current climatic balance.	Global SPEI dataset
Temperature	Environmental stress	The average maximum temperature for each province (measured in °C).	TerraClimate dataset

### Health outcomes and social determinants of child health

We retrieved two indicators of child health from the Children’s Institute database: low birth weight and severe acute malnutrition. Low birth weight (LBW) prevalence is measured by the proportion of children who weigh less than 2.5 kg at birth. Severe acute malnutrition incidence (henceforth malnutrition) is measured by the number of children under 5 years of age (under-5) newly diagnosed with severe malnutrition per 1000 under-5 children.

Additionally, we collected two variables related to parental care and child nutrition. Parental care was denoted by orphan status, which measures the proportion of children without both maternal and paternal parents. Child nutrition was noted by child food poverty (henceforth food poverty), which is measured by the proportion of children who reside in income-poor households. In this case, the food poverty indicator is used to determine the poverty status of the children, with households considered poor if they earn below the poverty line (i.e., less than R663 per month using 2022 as a reference point).

We collected three additional variables which measure the level of access to basic services related to health, such as clinics, water, and sanitation. Proximity to clinic is measured by the proportion of children living far from health facilities. Health facilities are considered to be far if they are more than 30 min away when considering the travel time of the household’s usual means of transport. Water access measures the proportion of children with access to safe and reliable drinking water at home. Sanitation access measures the proportion of children who reside in a household with access to basic sanitation. Access to adequate toilet facilities is used as an indicator of basic sanitation. Overall, we have a consistent sample size throughout the study period with nine observations for each variable in 2007, 2012, and 2017.

### Environmental predictors

Three environmental variables were collected, each representing a distinct form of drought or environmental stressor. First, the Normalised Difference Vegetation Index (NDVI) data were collected to represent the extent and distribution of agricultural drought.

The NDVI is useful for measuring vegetation greenness, which can be used to track plant health over time. Vegetation health is linked to, among others, soil moisture, temperature, and humidity, all of which are key indicators of drought^56^. Therefore, the sensitivity of NDVI to these factors, and its suitability for tracking large-scale and long-term vegetation dynamics make it an ideal proxy for agricultural drought (See^56–58^. Second, the Standardised Precipitation Evapotranspiration Index (SPEI) data were collected at a 12-month timescale to represent hydrological drought and its impact on child health. Last, maximum temperature data were collected to represent environmental stress. All these indices were retrieved as annual averages for each province from 2002 to 2022. The timeline was selected to match the temporal extent of the child health indicators and social determinants.

### Statistical analyses

Prior to the analysis, LBW, orphan status, sanitation access, water access, and food poverty variables were divided by 100 so the values can be bound between 0 and 1, making it suitable for a beta regression^59^. Then, the following analyses were conducted in R 4.4.2^60^. First, we fitted three beta regression models to individually investigate the predictors of LBW, sanitation access, and water access (Models 1–3). A beta regression model is suitable for modelling proportion data, as explained above^59^. A one-year lag was applied to LWB to account for the time dependence of environmental impacts. Then, we fitted a negative binomial model to investigate the socio-environmental predictors of malnutrition (Model 4). The negative binomial model was chosen for its ability to handle count data with extensive overdispersion^61^. Then, we fitted a Gaussian model to investigate the predictors of SPEI, which is normally distributed (Model 5). Thereafter, we combined the five models into a structural equation model (SEM) to elucidate the pathways through which the socio-environmental factors predict indicators of child health. The model was fitted using the *piecewiseSEM* package^62^, and Fisher’s *C* statistics was used to assess the model fitness, with a p-value greater than 0.05 indicating an acceptable fit to the data.

## Data Availability

Data used in the analysis are available online. The child health data were retrieved from the Children’s Institute (https://www.datafirst.uct.ac.za/dataportal/index.php). The Google Earth Engine Platform (https://earth.google.com/web/) was used to retrieve drought-related data from various databases: NDVI (https://modis.gsfc.nasa.gov/data/dataprod/mod13.php), SPEI (https://spei.csic.es/), and temperature (https://www.climatologylab.org/terraclimate.html).

## Code availability

The code associated with the analyses is available online^63^.
